# Developing Effective and Efficient care pathways in chronic Pain: DEEP study protocol

**DOI:** 10.1186/1472-6831-14-6

**Published:** 2014-01-21

**Authors:** Justin Durham, Matthew Breckons, Vera Araujo-Soares, Catherine Exley, Jimmy Steele, Luke Vale

**Affiliations:** 1 Institute of Health & Society, Newcastle University, Baddiley-Clark Building, Richardson Road, Newcastle upon Tyne NE2 4AX, UK; 2 Centre for Oral Health Research (COHR), Newcastle University, Newcastle upon Tyne, UK

**Keywords:** Orofacial pain, Health economics, Quality of life, Qualitative methods, Chronic pain, Care pathways

## Abstract

**Background:**

Pain affecting the face or mouth and lasting longer than three months (“chronic orofacial pain”, COFP) is relatively common in the UK. This study aims to describe and model current care pathways for COFP patients, identify areas where current pathways could be modified, and model whether these changes would improve outcomes for patients and use resources more efficiently.

**Methods/Design:**

The study takes a prospective operations research approach. A cohort of primary and secondary care COFP patients (n = 240) will be recruited at differing stages of their care in order to follow and analyse their journey through care. The cohort will be followed for two years with data collected at baseline 6, 12, 18, and 24 months on: 1) experiences of the care pathway and its impacts; 2) quality of life; 3) pain; 4) use of health services and costs incurred; 5) illness perceptions. Qualitative in-depth interviews will be used to collect data on patient experiences from a purposive sub-sample of the total cohort (n = 30) at baseline, 12 and 24 months. Four separate appraisal groups (public, patient, clincian, service manager/commissioning) will then be given data from the pathway analysis and asked to determine their priority areas for change. The proposals from appraisal groups will inform an economic modelling exercise. Findings from the economic modelling will be presented as incremental costs, Quality Adjusted Life Years (QALYs), and the incremental cost per QALY gained. At the end of the modelling a series of recommendations for service change will be available for implementation or further trial if necessary.

**Discussion:**

The recent white paper on health and the report from the NHS Forum identified chronic conditions as priority areas and whilst technology can improve outcomes, so can simple, appropriate and well-defined clinical care pathways. Understanding the opportunity cost related to care pathways benefits the wider NHS. This research develops a method to help design efficient systems built around one condition (COFP), but the principles should be applicable to a wide range of other chronic and long-term conditions.

## Background

Chronic pain is a distressing problem for patients and is difficult, and sometimes distressing, to manage for clinicians [1-4]. Chronic orofacial pain (COFP) affects a reported 13% of the UK population, and is particularly complex and distressing for patients [5-9]. Diagnosis and treatment for COFP conditions is slowly improving through the institution of new, targeted, diagnostic tools [10] and advances in genomics [11], but current care pathways do not seem to maximise therapeutic potential and paradoxically may worsen COFP [6,12].

COFP patients are known to use more healthcare resource compared to other dental patients [13-17], but what is unknown is why, or where, this utilisation occurs and how effective it is. Previous research [1,6,12,13] seems to suggest that a large proportion of this resource utilisation may occur as a result of inadequate care pathways for patients with COFP: cyclical referrals accompanied by multiple and unnecessary consultations which often only serve to increase confusion and sometimes worsen the patient’s complaint [6]. This is a costly process for both the patient and the health service and therefore in addition to delivering more accurate diagnoses and treatment there is an urgent need to understand how and where services can be streamlined in order to get patients to the most appropriate care effectively and efficiently.

A simplistic unidimensional assessment of the costs of care pathways is insufficient to capture the biopsychosocial dynamic relationship of COFP and the care received [18]. A “whole systems perspective” [19] is required in order to assess current care pathways and produce patient centred services “designed around patient’s needs” [20]. This will help achieve one of the recent recommendations of the recent national pain audit in the UK, which is to “research…optimal models of care for people with chronic pain, including economic modelling” [21]. Without identifying where the negative economic, biomedical, and psychosocial impacts exist on the current care pathway from both the consumer and the providers’ perspectives, it is impossible to model new pathways that provide appropriate care in a patient-centred, efficient, efficacious and expedient manner.

### Aims and objectives

This study will describe and model current care pathways for COFP patients, identify areas where the current pathways could be modified, and model the estimated impact of change to determine what changes would improve outcomes for patients and use resources more efficiently.

Specifically it will:

Phase 1

i) Develop a map of COFP patients’ journeys through care and understand their experiences of the care pathway using qualitative in-depth interviews.

ii) Identify the impacts of the various stages of care pathways on: individual’s pain (West Haven Yale Multidimensional Pain Inventory, WYMPI [22]; Graded Chronic Pain Scale, GCPS [23]); quality of life, (EQ-5D) and the value that patients attach to the various stages of their care pathway [24]; use of health service and patient costs (use of health service and patient costs questionnaire [25]); illness perceptions (Revised illness perceptions questionnaire, IPQ-R [26]).

Phase 2

iii) Develop a model based upon the care pathways reflecting key events (e.g. referrals, use of services, impact on pain and daily living) and use this model to estimate the cost and outcomes (e.g. level of pain, quality of life).

iv) Use the data gathered from objectives i to iii and work with stakeholders, to identify priority areas where the current pathway might be changed and model the impact of the potential changes on costs, outcomes and cost-effectiveness of care for COFP.

v) Use the results of (i-iv) above develop recommendations for practice and future research

## Methods/Design

**Figure 1:**
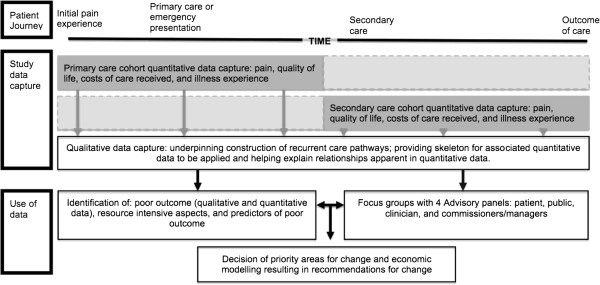
Schematic of patient journey, data capture, and use of data from study.

We propose to capture data across the journey through care for COFP patients. This journey will start with the experience of pain and the individual will seek help from the health service, most frequently through primary care, but not exclusively so. The care journey may, for a minority, be a short one, but the current literature suggests that multiple healthcare providers may be consulted about the condition and the journey may not be as linear as depicted by Figure 1[6,12,13,21]. If this were the case it would mean that to capture the entirety of some individuals’ care pathway(s) might take several years, which is impractical for a number of reasons. This study proposes to solve this problem by purposively sampling individuals suffering from COFP along the continuum of care from initial experience of pain through to a final outcome which may be successful treatment, or the acknowledgement no more can be done (Figure 1). In this manner we will be able to capture data from all aspects of the care journey and pathway for two years, which will result in some individuals describing whole journeys (Figure 1 – dark plus light grey boxes) and some describing particular aspects or points of the care pathway (Figure 1 – dark grey boxes).

This data collection across the continuum will allow a complete picture of the possible care pathways for COFP patients to be built. Data collection will be accomplished through qualitative and quantitative methods with the qualitative data helping explain any apparent relationships in the quantitative data.

## Phase 1 – Recruitment, observation, recording, and mapping the current pathway(s)

### Methods

**Figure 2:**
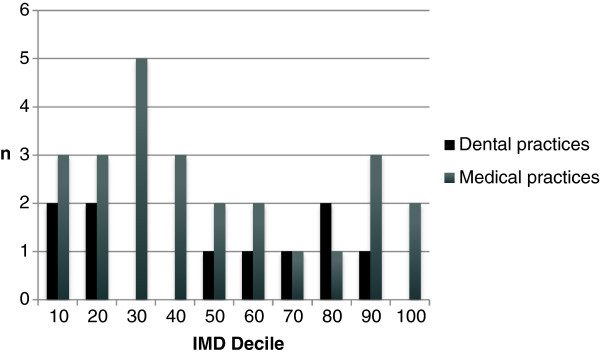
**Sociodemographic of practices involved in study.** This figure is calculated by using the 2010 UK census data available at http://neighbourhood.statistics.gov.uk/dissemination/. This census defined 32482 small geographic areas in England each consisting of approximately 1500 people (Lower super-output areas [LSOA]). Each LSOA was assessed and scored according to standardised criteria across 38 domains and then ranked from the best score (rank 1) to the worst (32482). These 38 domains included income, health, and employment [27] and were combined to produce a composite “index of multiple deprivation” (IMD) which was ranked in the same manner. The deciles in the figure above represent the rounded percentage ranking with lower deciles equating to worse deprivation according to IMD score.

Patients will be recruited from Primary and Secondary care in the North East of England. Primary care recruitment will take place from 25 medical practices and 10 dental practices from diverse socioeconomic areas (Figure 2). Secondary care patients will be recruited from a variety of clinics in the local dental and medical hospitals: neurology, oral and maxillofacial surgery, dental emergency clinic, oral medicine, and restorative dentistry.

Using a total sample size of 200, with a Type I error of 5%, we can, with 80% power, detect an effect size of 0.4 within our data (Two-tailed). This represents a moderate effect size [28] on which to base our sample size because a smaller effect size, if detected, would be unlikely to mandate significant changes in the healthcare system. Allowing for 20% attrition provides a final sample size of 240. This attrition rate is realistic because of our previous experience with longitudinal data collection with patients suffering from COFP showing a high dropout rate.

### Inclusion and exclusion criteria

Participants will be over the age of eighteen. Orofacial pain will have been present for greater than or equal to three months [29]. Using a validated dual baseline-screening questionnaire (BSQ1) [30,31] the participants will be categorised post-initial recruitment to assign their orofacial pain to a neurological/vascular (from nerves or blood vessels), Dentoalveolar (from tooth or tooth bearing structure), or Temporomandibular disorder/musculoskeletal cause. The sample will be stratified by care sector and gender.

Where a specialist clinical diagnosis is available which suggests a negative screening result from the BSQ1 is a false negative the Chief Investigator (CI) will review the sub-diagnosis automatically generated by the screening questionnaire along with the individual’s clinical diagnosis. The CI will then only include the patient in the study if the sub diagnosis or specialist clinical diagnosis is part of the group of conditions being studied: all types of headache, temporomandibular disorders (TMDs), neuralgias, burning mouth syndrome, traumatic neuropathies, and persistent dentoalveolar pain disorder (atypical odontalgia).

The exclusion criteria are that: 1) an individual lacks the capacity to give informed consent for any reason; 2) an individual is categorised by the screening questionnaire as *only* having dentoalveolar pain, which is not part of the group of conditions that comprise COFP; 3) an individual is unable to communicate complex constructs in English given the qualitative aspects of the overall study.

### Recruitment

The participating clinical and or research team will both prospectively and retrospectively identify individuals eligible to be included in the study on the initial basis of: their age, the duration of their complaint, and presumed diagnosis. Electronic and paper adverts advertising the study will also be placed within the practices participating and any allied clinical facilities such as pharmacies they use. Adverts will also be placed in public places and in the local press if necessary. The advert provides the contact details for the study team and, if appropriate, the individual contacting the study team will be recruited as described below.

For prospective recruitment the research or clinical team will give the patient a short standardised verbal description of the study and ask if the patient is interested in being involved. Standardised recruitment pro forma will be used to record those who are interested in participating in the study and those who decline in order to facilitate an analysis of both those who decline and eventually those who fail to respond or complete the study. Those who are interested in participating will be issued with a phase 1 patient pack, which includes: a patient information sheet, phase 1 consent form and the BSQ1.

For retrospective recruitment standardised letters from the patient’s primary care practitioner explaining the study will be sent out to patients seen within the last year with pain fitting the inclusion criteria to ascertain if they are interested in taking part in the study. Patients contacted in this manner will be asked to contact the research team if they are interested in participating and then the research team will give a standardised verbal explanation of the study by phone, complete the recruitment proforma, and then send out the phase 1 patient pack.

In both prospective and retrospective recruitment, a trained member of the research team will contact those interested within the next fortnight by telephone, or at their next clinic appointment, in order to complete the BSQ1 verbally if the individual is still willing to participate. The BSQ1 is completed by the researcher in accordance with the participant’s responses within Excel (Excel v10, Microsoft Corporation, Redmond, WA, USA) and gives an immediate outcome in relation to the inclusion criteria. Those screening positive and giving informed consent, will then be enrolled in the study. Those who withhold consent will be thanked for their interest and will not be enrolled. Those who screen negative will be thanked for their interest and will not be enrolled unless a false negative is suspected whereby the CI will review all available results for the individual. Those enrolled in the study, after screening positive and giving consent, will be contacted by the research team at a time and location convenient to them to conduct baseline data collection. Any withdrawals from the enrolled cohort will be noted along with age, gender, BSQ1 classification, and the broad reason given for their withdrawal.

### Data collection

All participants (n = 240) will complete a baseline structured interview (Case report form [CRF]) with a trained member of the research team, either by phone or face-to-face, in order to: a) capture baseline sociodemographic data; b) capture data about their pain to that point (duration, treatment received and its effectiveness, healthcare practitioners seen); c) ensure their comprehension of the instruments to be used over the next two years.

**Figure 3:**
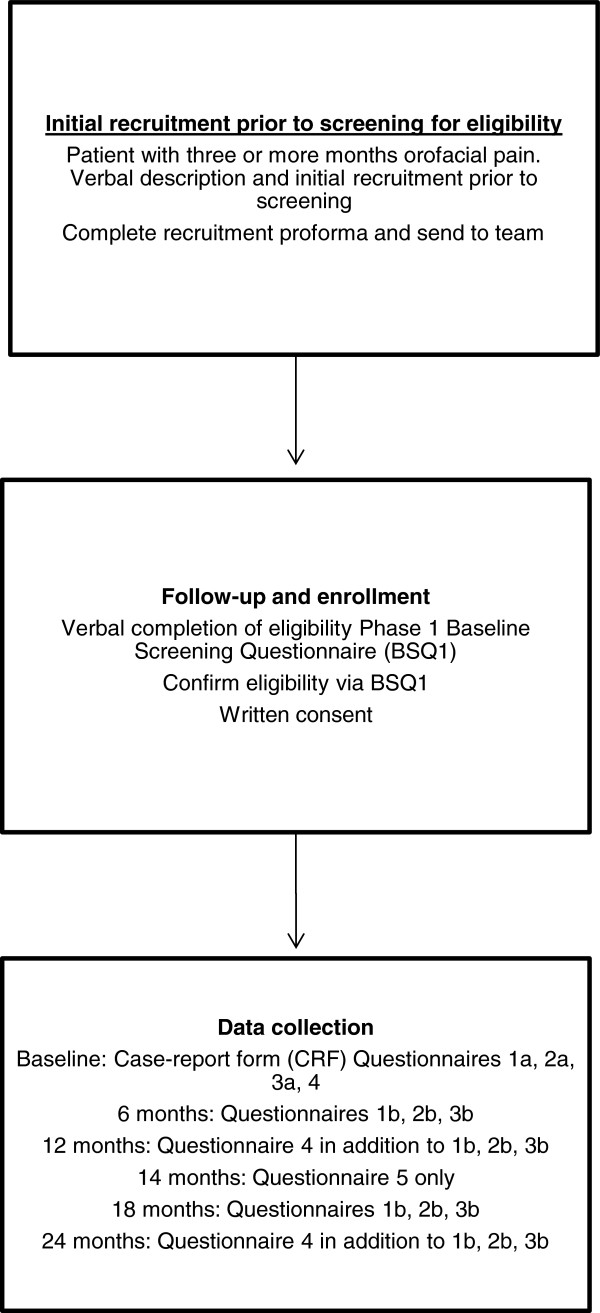
Study flowchart for Phase 1.

We have identified the impact of pain, quality of life, and costs as the three most appropriate measures of success in a healthcare system attempting to manage COFP. The instruments used to gather quantitative data on the impact and degree of pain, quality of life, and costs of illness at the varying data collection points (Figure 3, Table 1) will be:

• A quality of life instrument will be issued at each of the six monthly data collection points. This will be the EQ-5D-5 L [24]). At baseline two reference periods will be used for the EQ-5D “last month” and “today” (Questionnaire 1a), thereafter only the standardised reference period of “today” will be used (Questionnaire 1b).

• Multidimensional pain measures – those used will be the Graded Chronic Pain Scale (GCPS) [32] and the West-Haven Yale Multidimensional Pain Inventory (WYMPI version 3) [22]. The baseline questionnaire 2a contains both the GCPS and the Pain impact and Spousal interactions subscales of the WYMPI, but questionnaire 2b used sequentially thereafter omits the final subscale of the WYMPI relating to spousal interactions in order to reduce respondent burden.

• Cost of illness instruments – To reduce respondent burden we will issue a “Use of services and productivity” questionnaire at each six monthly data collection points (Questionnaire 3) and a one-off “Time and Travel” questionnaire (Questionnaire 5) [25] at fourteen months into the study. Questionnaire 3 has two versions in order to try and reduce respondent burden: version “a” for administration at baseline, and version “b” for sequential administration thereafter. Version “b” is almost identical to the “a” version other than the omission of some questions that cannot change from baseline for example, “occupation when pain started”, and giving options to record sections as “no change” since last administration. Illness perceptions, anxiety and depression will also be briefly examined in order to help profile the study cohort. This will be accomplished through using the IPQ-R and PHQ-4 in questionnaire 4 which will be issued at baseline, twelve and twenty-four months [26,33-36].

**Table 1:** Phase 1 questionnaire administration timetable

	**Issued at:**						
**Questionnaire**	**Recruitment**	**Baseline**	**6 M**	**12 M**	**14 M**	**18 M**	**24 M**
BSQ1	X						
CRF		X					
EQ-5D (Q1a)		X					
EQ-5D (Q1b)			X	X		X	X
GCPS & WHYMPI (Q2a)		X					
GCPS & WHYMPI (Q2b)			X	X		X	X
Baseline use of services and productivity (Q3a)		X					
Ongoing use of services and productivity (Q3b)			X	X		X	X
IPQ-R and PHQ-4 (Q4)		X		X			X
Time and travel questionnaire (Q5)					X		

A notes-based analysis of consultation, prescription, and referral histories will supplement the self-complete data where necessary and telephone interviews will be held with patients whose data requires further clarification. Throughout the quantitative data collection interim analyses will be conducted on the immature data sets to help identify and explain missing data, and shift the analysis burden.

Any individuals whose data has not been received fourteen days after the instruments were posted to them will be followed up using a standardised operating protocol: reminder letter, then telephone call, and then contact with nominated secondary point of contact if none of the previous modalities of contact have been successful in reaching the individual or their voicemail. An individual will be assumed to have withdrawn in absentia ten days after leaving a message with a voicemail or contacting the secondary point of contact; should they subsequently contact the research team and express a desire to continue all efforts will be made, as far as reasonably practical, to facilitate this.

Qualitative interviews will take place with a purposive sub-sample (n = 30) of the total cohort. The sub-sample will be taken using: gender; strata for time in care; type of care environment; origin of COFP (Table 2). Telephone or face-to-face qualitative interviews will be conducted at baseline with this sub-sample by trained and experienced interviewers using a flexible evolving topic guide. It is planned that the same sub-sample will be interviewed again at twelve months and twenty-four months by telephone or face-to-face in order to examine any further experiences or altered perceptions. Data collection and analysis will follow the principles of the constant comparative method [37] whereby data collection and analysis occur concurrently and continue until saturation, which will allow us to add to our sample should interesting issues arise. The interviews aim to build an understanding of: the illness the individual is experiencing, the journey(s) through care, and any apparent relationships in the quantitative data.

**Table 2:** Purposive subsampling criteria

**Stratification**	**Details**
Gender	Attempt to gain a 1:1 ratio of gender in subsample
Care environment	Attempt to gain a 1:1 ratio of those in primary care at baseline and those in secondary care at baseline
Time in care	Attempt to gain a subsample that contains three broad groups:
	First experience of COFP (maximum 6–12 month history)
	Moderate experience of COFP (13–23 month history)
	Long experience of COFP (>23 month history)
Origin of COFP	Attempt to get an equal representation of musculoskeletal and neuropathic/vascular origins in sample

### Analysis

Data on use of services (Q3a, 3b and 5) will be used to calculate costs by combining information on resource use with unit costs either developed as study specific estimates or obtained from routinely available sources for example the unit costs of health care [38], the British National Formulary for medications [39]. The EQ-5D 5 L responses will be converted into health state utilities using tariffs currently under development or cross-walked to the pre-existent EQ-5D-3 L UK population health tariffs [40,41] and will estimate quality adjusted life years (QALYs [42]).

Data from GCPS and WYMPI (Q2a and 2b) will be used in order to build a sequential multidimensional picture of the pain’s impact on individuals. As GCPS has been shown to be predictive of outcome in one specific type of COFP (TMDs [23]) it will be examined specifically to see if it provides a good predictor of outcome in primary and or secondary care. The omission of the spousal interactions section from WYMPI after baseline administration will mean that this cannot be examined in relation to change, but it will help categorise our sample at baseline.

The IPQ-R will give us sequential data on the patient’s lay perception of their symptoms’: identity, cause, severity, consequences, treatment/control. This instrument will also allow us to assess at defined points during their care pathway the emotional impact of their illness as well as their understanding (perceived coherence) of their illness.

Scores from the questionnaires are likely to need to be transformed to allow one-way and repeated ANOVA to determine if there are significant differences in cost across quality of life scores and pain. Follow-up regression analysis will then be used to determine the multivariate predictors of these differences including GCPS, care sector, education, and other sociodemographic variables. This data analysis will then help inform the appraisal groups.

## Phase two methods: pathway analysis and priority setting

The aims of Phase 2 are:

1) To develop a model based upon the care pathways reflecting key events (e.g. referrals, use of services, impact on pain and daily living) and use this model to estimate the cost and outcomes (e.g. level of pain, quality of life).

2) To use the data gathered and work with stakeholders, to identify areas where the current pathway might be changed and model the impact of the potential changes on costs, outcomes and cost-effectiveness of care for COFP.

### Methods

The analysis of the qualitative data will be used to assemble the framework (map) of current experiences of the journey through care using an iterative methodology to record recurring emergent experiences against the generic stages of the patients’ journeys [6]. The qualitative data will be used to highlight any areas of concern for the sample on the map of the current journey through care with the quantitative data used alongside the qualitative data to help quantify the degree of impact and identify areas that, if changed, may produce the most benefit to the patients and health service. This will be an iterative process that will produce an understanding and inform a model that will be developed to describe the care pathways experienced.

The map and quantitative longitudinal data will in themselves illustrate areas of high cost and poor outcome (pain, quality of life) but the discussion on which areas of the pathway are a priority to change will be conducted with four separate appraisal groups: public, patient, clinician, and commissioning and managing groups, each consisting of 5–8 members of a variety of ages from the local area. COFP patients and the public (lay) participants will be recruited from routine diagnostic clinics in the Newcastle-Upon-Tyne Hospitals Trust using a standardised PIS and consent form. The inclusion and exclusion criteria for patients will mirror that of phase 1 using the same screening questionnaire. To be accepted as a member of the public the following inclusion criteria must be met through responding to a short screening questionnaire:

• The individual in question nor their family have on-going pain in their mouth and or face within the last twelve months

• The individual in question nor their family are health professionals

A standardised letter will be used to approach local clinicians and service managers/commissioners at their respective professional addresses. The letter will contain the Patient Information Sheet (PIS) and consent form and contact details for the research team.

The appraisal groups will be presented with the study findings in a comprehensible annonymised form two weeks prior to convening and upon convening will be conducted in a focus group manner. The groups will be recorded and transcribed verbatim, helping guide the decisions on the priority areas in the current care pathway to change in the subsequent economic modelling. Trained facilitators using flexible and evolving topic guides will undertake the focus groups. The groups will be digitally recorded and transcribed verbatim. Qualitative analysis of the focus group data will follow the same principles as those described in Phase 1.

## Phase three methods: economic modelling and recommendations

The aim of phase three is:

1) Using the results of phase one and two, develop recommendations for practice and future research

This phase consists of developing an economic model based upon the care pathways determined in phases 1 and 2. The model will describe the logical and temporal sequence of events from first presentation with COFP in primary care through any subsequent management in both primary and secondary care. The model will be developed in line with best practice [43]. We anticipate that the model may take the form of a Markov model but the precise form of the model will be determined as part of the project and will be chosen to fit the processes modelled. The outputs of the model will be cumulative costs and QALYs over a 5-year period (i.e. the time period over which we believe data can reliably extrapolated) but we will explore in a sensitivity analysis the impact of conducting a longer (e.g. lifetime) time horizons. The perspective will be that of the UK NHS and patients and discounting in the base case will be at 3.5% [44].

The parameter estimates (probabilities, costs and utilities) required for the model will come from the longitudinal study described above, focused searches of the literature and advice from an expert panel. All uncertainty surrounding estimates of input parameters will be informed by appropriate distributions calculated from the longitudinal study or the literature. The results of the economic model will be presented as incremental costs and QALYs, and the incremental cost per QALY gained. Both deterministic and probabilistic sensitivity analyses will be carried out to test and explore uncertainties. The results of the probabilistic sensitivity analysis will be presented as a series of cost-effectiveness acceptability curves (CEACs). At the end of the modelling process recommendations for service change will be available for evaluation and further research.

### Ethical approval

This study has approval from the Yorkshire and the Humber (Leeds West) Ethics Committee (Ref: 12/YH/0338) and NHS R&D approval from each participating site. Honorary NHS contracts have been issued where necessary.

## Discussion

Currently there are data, which suggest that patients with COFP use a large amount of healthcare resource [13-17]. What is unknown, and this study seeks to identify, is if the resource that is used is proportional and effective for their complaint?

It is conceivable that the only reason COFP patients use so much resource is because the care system they experience fails to provide them with clear and defined pathways of care based upon early diagnosis and appropriate management. The need for early diagnosis and management is key given that those with a propensity for developing psychological comorbidities may develop these sooner if diagnosis is delayed or there is a misdiagnosis causing uncertainty or anxiety over the nature of the complaint [5,45,46]. Any psychological comorbidities that develop will then negatively impact on their prognosis [23,47-49].

Early appropriate conservative management [50] is also important given the emerging role for central sensitisation [51-57] and the autonomic nervous system (ANS) in COFP [58]. Reducing the peripheral afferent barrage at the earliest opportunity and down regulating any dysfunctional ANS as soon as possible through early diagnosis, reassurance, and management will also all hopefully reduce the chance of central neuroplastic changes, “central sensitisation” [51,59]. Reducing the potential for central sensitisation, or up regulation of the ANS, occurring may then help improve the success of (simpler) therapies, reduce treatment times, and improve prognosis by reducing the potential for the condition becoming chronic.

There are clear and evidence-based methods for managing generic chronic pain in an interdisciplinary fashion of which a substantial proportion may be translatable into COFP [60,61]. A recent national pain audit [21] in the U.K. has, however, highlighted the difficulty in establishing the provision of such a service for generic chronic pain management nationwide, irrespective of the need for a condition specific service. The same audit also highlighted that despite the evidence base supporting interdisciplinary pain management, it is still unclear how to clinically and cost effectively provide (NHS) healthcare services for patients with chronic pain [21]. Against the background of changes in commissioning and the drive to provide services more cost-effectively in the NHS [62] the DEEP study may provide answers for COFP care pathways and also a model and methodology by which to examine other long-term conditions’ care pathways.

## Abbreviations

ANS: Autonomic nervous system; ANOVA: Analysis of variance; BSQ1: Baseline screening questionnaire completed in phase 1 prior to enrolment; CI: Chief investigator; CEAC: Cost effectiveness acceptability curve; COFP: Chronic orofacial pain; CRF: Case report form; DEEP: Developing Effective and Efficient care Pathways in chronic pain; GCPS: Graded Chronic Pain Scale; IPQ-R: Revised illness perceptions questionnaire; NHS: National Health Service in UK; PHQ-4: Patient health questionnaire 4; PIS: Patient information sheet; QALYs: Quality adjusted life years; TMDs: Temporomandibular disorders; WYHMPI: West-haven Yale Multidimensional Pain inventory.

## Competing interests

The authors declare that they have no competing interests.

## Authors’ contributions

JD, MB, CE, JGS, VAS, LV were all involved in original conception and design of the study. All authors helped draft this manuscript and were involved in revising the manuscript critically for important intellectual content and have given approval of the final manuscript.

## Authors’ information

JD is a Senior Lecturer in Oral Surgery and Orofacial Pain and NIHR Clinician Scientist. MB is a Research Associate. VAS is a Senior Lecturer in Health Psychology. CE is a Senior Lecturer in Medical Sociology. JGS is a Professor of Oral Health Services Research. LV is a Professor of Health Economics.

## Pre-publication history

The pre-publication history for this paper can be accessed here:

http://www.biomedcentral.com/1472-6831/14/6/prepub
